# Supplementary material for: MetaRibo-Seq measures translation in microbiomes
Source: Nat Commun. 2020 Jun 29;11:3268. doi: 10.1038/s41467-020-17081-z (PMC7324362; doi:10.1038/s41467-020-17081-z)
Supplement: Supplementary file 10 — Supplementary Data 7 [file 41467_2020_17081_MOESM10_ESM.zip › File2/Confidence_High_Taxonomy/438555_out.krona.html]

Javascript must be enabled to view this page.

members
magnitude
magnitudeUnassigned
count
unassigned
taxon
rank

438555\_out

7

2
7
superkingdom

1224
7
phylum

7
1236
class

order
91347
7

543
7
family

genus
7
561

species

SRS017521\_contig\_number\_30110SRS049402\_contig\_number\_15737SRS076876\_contig\_number\_1203SRS098881\_contig\_number\_12820SRS144362\_contig\_number\_31487SRS146888\_contig\_number\_15617SRS147022\_contig\_number\_13110
7
562
